# Cell-Mediated Immunity Against Human Papillomavirus Infection: From Viral Clearance to Oncogenesis

**DOI:** 10.3390/v18030362

**Published:** 2026-03-16

**Authors:** Diana Savage, Jiafen Hu, Adam D. Burgener, Afshin Raouf, Thomas T. Murooka

**Affiliations:** 1 Department of Immunology, Rady Faculty of Health Sciences, University of Manitoba, Winnipeg, MB R3E 0T5, Canada; topolni3@myumanitoba.ca (D.S.); afshin.raouf@umanitoba.ca (A.R.); 2 Paul Albrechtsen Research Institute, CancerCare Manitoba, Winnipeg, MB R3E 0V9, Canada; 3 Department of Pathology and Laboratory Medicine, Pennsylvania State University College of Medicine, Hershey, PA 17033, USA; fjh4@psu.edu; 4 Center for Global Health and Diseases, Department of Pathology, Case Western Reserve University School of Medicine, Cleveland, OH 44106, USA; adam.burgener@case.edu

**Keywords:** human papillomavirus, mucosal infection, cutaneous infection, cell-mediated immunity, T cell response, immunotherapy

## Abstract

Human papillomavirus (HPV), especially high-risk HPV types, is a significant public health concern due to its association with various cancers and increased risk of acquiring other sexually transmitted infections (STIs). In most cases, host immunity rapidly responds to and clears HPV infections, but persistent or latent infections can increase susceptibility to cancer. A better understanding of how HPV interacts with and evades the immune response is vital to understanding disease progression and guiding the next generation of vaccines and immunotherapies. This review article provides a comprehensive overview of the immune mechanisms involved in HPV infection, highlighting the roles of T cells and other immune subsets. We discuss the immune evasion strategies employed by HPV and subsequent modulation of the immune microenvironment. Additionally, we explore the current therapeutic landscape and emerging immunotherapeutic approaches under investigation. By unveiling the intricacies of the immune response to HPV, we may inform improved strategies for the treatment of HPV-related diseases.

## 1. Introduction

Human papillomaviruses (HPVs) are a large and diverse family of small double-stranded DNA viruses that are divided into five genera: alpha (α), beta (β), gamma (γ), mu (μ), and nu (ν). Of these, the α- and β-HPVs have garnered the most attention due to their associations with human health [1,2,3,4,5,6]. Currently, more than 400 HPV types have been identified [7]. α-HPVs primarily infect the mucosal epithelium and are comprised of both low- and high-risk types based on their ability to cause malignant transformation of infected tissue. Broadly, high-risk HPV16 and HPV18 are the predominant drivers of virus-induced pathogenicity, linked to the development of oropharyngeal, anal, penile, cervical, vaginal, and/or vulvar cancers, whereas low-risk HPV6 and HPV11 are typically associated with benign genital warts [8]. β-HPVs primarily cause transient infections of the skin but are a frequent cause of skin cancers such as cutaneous squamous cell carcinoma (cSCC) in immunocompromised individuals [2,3,4]. The ability of HPV to establish chronic infection at these tissue sites has important implications for tissue pathology and cancer development and remains a key research area in the absence of an approved therapeutic vaccine. In this review, we provide an overview of how the cutaneous and mucosal tissues generate critical cell-mediated immune responses against HPV infection, with a focus on CD8^+^ and CD4^+^ T cell responses. Understanding protective immunity and HPV immune evasion can be leveraged to develop vaccine-based therapies against HPV^+^ cancers, with several approaches showing clinical promise.

## 2. Etiology and Epidemiology of HPV Infection

### 2.1. Pattern and Prevalence of HPV Infection and HPV-Induced Cancers

HPV is one of the most common sexually transmitted infections, with nearly all sexually active men and women acquiring it at least once in their lifetime [9]. It is predominantly transmitted through sexual contact, affecting tissues including the cervix and anal canal; however, HPV transmission may also occur perinatally [10,11,12]. Risk factors associated with HPV infection include multiple lifetime sexual partners, early sexual debut, and contact with high-risk individuals [10,12]. Subclinical HPV infections, during which infected cells retain normal morphology despite the presence of viral DNA measured by PCR analysis of cervical samples, can be detected in up to ~40% of sexually active women [12]. Despite frequent infection by HPV, 80–90% of infections are resolved spontaneously within two years post exposure. However, in 15–20% of women, HPV infection becomes persistent and contributes to a higher risk of developing neoplastic lesions of the anogenital tract [10]. Progression is impacted by viral type, viral load, prolonged use of oral contraceptives, and high parity [13,14]. In contrast, β-HPVs can either play a protective or tumor-promoting role, especially given the rising incidence of skin cancer worldwide. β-HPVs are common components of the skin’s commensal flora in more than 50% of individuals [15]. Notably, β-HPVs have been detected in more than 80% of SCCs in oral transplant recipients, suggesting a potential role in carcinogenesis. HPV-induced SCCs are associated with β-HPV types 5 and 8 in patients with epidermodysplasia verruciformis (EV), transplant recipients, and even healthy individuals. Although pathogenic β-HPV infection could induce cutaneous lesions and warts in the general population, carcinogenesis caused by β-HPV is more frequently associated with environmental factors and host immune status [5,15].

### 2.2. Etiology and Pathogenesis of α-HPV and β-HPV Infections

Most current knowledge of etiology and pathogenesis has been derived from α-HPVs and may also be applicable to some β-HPVs. The 8 kilobase pair, double-stranded, circular DNA genome of HPVs consists of an upstream regulatory region (URR), an early (E) gene region, and a late (L) gene region [8]. The URR contains the binding sites for various cellular transcription factors and the two early viral proteins (E1 and E2) that are responsible for initiating viral replication and transcription within the host [8]. The early region encodes viral proteins E1, E2, E1^E4 (E4), E5, E6, and E7, while the late gene region encodes L1 and L2 [8]. HPV virions access basal keratinocytes through microabrasions in the epithelium, where the L1 major capsid protein binds heparin sulfate proteoglycans on the basement membrane, exposing the L2 minor capsid protein [16,17]. Subsequent conformational rearrangements of L1 and L2 promote internalization by endocytosis [16,17]. Upon entry, the HPV genome is transported to the nucleus and may be integrated into the host genome or maintained as a non-integrated element that replicates alongside the host genome, known as episomal DNA [18]. As the basal keratinocytes divide, two equally infected daughter cells are produced, one of which remains in the basal layer, establishing a reservoir of persistent infection, while the second migrates outward through the epithelium [7,19]. Partitioning of viral genomes to daughter cells is mediated by early protein E2 [20]. As the cells migrate, viral proteins are sequentially expressed, allowing for timely and efficient genome replication, packaging, and virion assembly. E1 and E2 recruit host cell machinery and initiate viral DNA replication and transcription [20,21]. Oncoproteins E5, E6, and E7 reprogram epithelial cells into a state of sustained proliferation and induce transformation [18,22]. Finally, capsid proteins L1 and L2 are synthesized within terminally differentiated epithelial cells, where they facilitate the packaging of newly synthesized DNA and viral transmission [23]. E1^E4 (E4) also disrupts the host cytokeratin network to facilitate viral egress [8]. Once formed, virions are released into the environment in dead cells that are sloughed off from the surface of the epithelium [7,19].

β-HPVs promote cellular proliferation in the presence of constant stress, such as ultraviolet (UV) radiation, enabling the accumulation of DNA damage [4]. In instances where the genes of β-HPV-targeted cellular pathways (e.g., p53) acquire mutations, the expression of viral oncogenes becomes irrelevant for the growth of cancer cells, as described through the hit-and-run hypothesis [4,24,25]. Interestingly, β-HPVs may also play a role as commensals with the ability to protect the host against virus-induced carcinogenesis [26]. The distinct mechanisms through which α- and β-HPV proteins interact with host cellular machinery and modulate the immune response may underlie their divergent pathological outcomes [27]. For instance, the oncoproteins E6 and E7 are essential for supporting persistent infection by both α- and β-HPV through the disruption of cellular apoptosis, differentiation, and DNA repair systems to ensure continuous cell proliferation and replication of the HPV genome [27,28]. Although the α- and β-HPV E6 and E7 proteins target many of the same pathways, including p53, BAK/BAX, and hTERT, they engage these pathways in distinct ways [27]. For example, α-HPV E6 directly binds and sequesters p53 whereas β-HPV E6 indirectly impacts its activity by preventing its phosphorylation by HIPK2 [27]. Furthermore, α- and β-HPVs differ in their expression of E5, which is found among the α-HPV types but is lacking in the β-HPVs. In the alpha genus, this protein has been shown to play a key role in the viral life cycle and in promoting tumor cell motility and metastasis [29,30]. Torres et al. infected *K14E5* transgenic mice, which express the HPV16 E5 gene in stratified epithelial cells, with mouse papillomavirus type 1 (MmuPV1) to investigate the effects of E5 on virus-induced pathogenesis [31]. Interestingly, they found that the skin lesions in these mice had an earlier onset, higher incidence, and reduced frequency of spontaneous regression compared to non-transgenic mice [31]. These findings underline a potential role for E5 in α-HPV driven oncogenesis. While uncovering the etiology of HPV provides insight into the underlying mechanisms of disease, examining viral spread dynamics and dysregulation of signaling pathways within target populations will further uncover a broader understanding of tissue-specific pathology. Key differences between α- and β-HPVs are highlighted in Table 1.

## 3. Interplay Between HPV Infection and the Immune Response

### 3.1. Comparing Transient, Acute, and Persistent HPV Infection Kinetics

Upon entry, HPV targets the basal layer of the epithelium, where it begins its replication cycle [32]. Here, viral proteins are produced and new viral particles are assembled using the host transcriptional and translational machinery. This process can cause infected cells to proliferate abnormally, leading to the formation of warts or other lesions [33]. HPV infections can be transient, acute, persistent, or commensal depending on various factors. Transient infections are typically self-limiting and cleared by the host immune response without long-term complications. Infections by non-oncogenic (or low-risk) α- and β-HPV types are largely asymptomatic [34]. Similarly, acute HPV infections are typically short-lived and cleared within months to years [35]. Unlike transient infections that are mostly asymptomatic, acute HPV infections often present with warts (papillomas) on the skin or at mucosal surfaces [36]. In some cases, acute HPV infections may appear to clear, but the viral genome persists in infected cells without detectable activity, establishing a latent infection that can be reactivated after a prolonged period—typically triggered by immunosuppression [36,37]. Acute infections, especially those caused by the high-risk HPV types (e.g., HPV16 and HPV18), can develop into persistent infections that have the potential to significantly increase the risk of developing precancerous lesions. Lesion formation is influenced by factors such as viral load and HPV type, environmental conditions, and host immune status, as discussed below [38]. The features that distinguish HPV infection states are highlighted in Table 2. Both innate and adaptive immune responses play essential roles in maintaining immunological homeostasis, particularly during pathogenic challenges. This review will focus on T cell-mediated immunity, which is critical for the elimination of HPV-infected cells [39].

### 3.2. Generation of Host Immunity to HPV Infection at Cutaneous and Mucosal Sites

**Figure 1:**
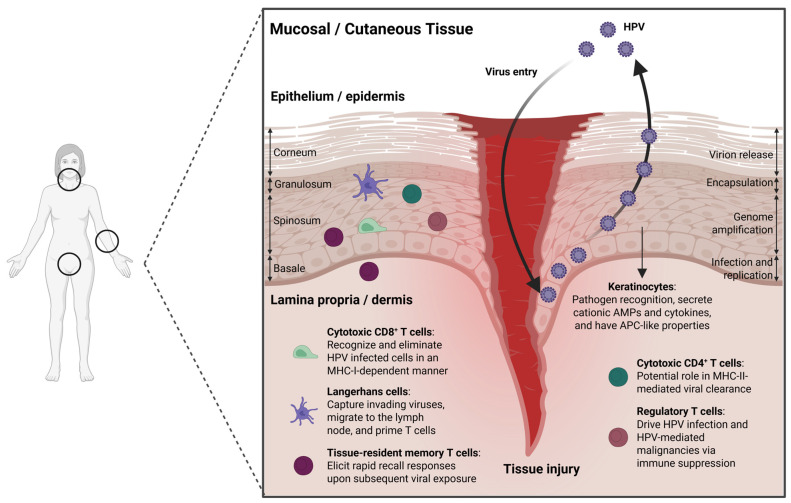
Overview of HPV infection dynamics within mucosal and cutaneous tissue. Upon entry, HPV infection is established in the stratum basale, a single layer of keratinocytes. As the virions are formed and mature, they move upward through the epithelium/epidermis and are ultimately shed from the stratum corneum. Within the epithelium/epidermis, cells such as keratinocytes, Langerhans cells, and cytotoxic CD8^+^ and CD4^+^ T cells contribute to viral clearance, while regulatory T cells contribute to persistence. Persistent viral infections may contribute to lesion formation and malignant transformation of epithelial cells. *Created in BioRender. Savage, D. (2026). BioRender.com/epgeky5*.

The skin and mucosal surfaces share anatomical similarities, with both the epidermal layer of the skin and the epithelial layer of the mucosa comprised of the stratum corneum, stratum granulosum, stratum spinosum, and stratum basale (Figure 1) [40]. The stratum basale is responsible for renewing the cells of the epidermis or mucosal epithelium and is comprised of a single layer of undifferentiated epidermal cells, known as keratinocytes. Basal keratinocytes mature as they move outward, ultimately reaching the stratum corneum, where they reside as terminally differentiated cells that function primarily in barrier protection and fluid loss [40]. Lineage tracing studies in mice identified CD271^+^Axin2^+^ basal stem cells responsible for epithelial regeneration and homeostasis and demonstrated their ability to reform the multilayered vaginal epithelium in 3D organoid cultures [41]. Importantly, keratinocytes participate in immune responses in the skin and mucosal tissues through the expression of toll-like receptors (TLRs), release of cationic antimicrobial peptides (AMPs) and inflammatory cytokines, and through their antigen-presenting cell (APC)-like activity (Figure 1), albeit limited in comparison to professional APCs [40,42,43,44]. Keratinocyte secretion of AMPs such as β-defensins and cathelicidins supports immune defense by recruiting antigen-presenting cells to infection sites, enhancing phagocytosis, modulating the cytokine environment, and promoting T cell recruitment and polarization [45]. These critical functions of keratinocytes are disrupted by HPV oncoprotein E7, which reverts terminally differentiated cells back into the S-phase to promote proliferation by interfering with tumor suppressor retinoblastoma protein (pRb) and E2F transcription factor interactions [46]. Furthermore, HPV-infected keratinocytes also downregulate their expression of viral proteins to limit antigen presentation during early phases of infection [47]. Viral E6/E7 proteins also interfere with the type I interferon response within infected keratinocytes. E6 can bind directly to Interferon Regulatory Factor 3 (IRF3), preventing its translocation to the nucleus and downstream transcription of IFN-β [48]. Likewise, both E6 and E7 have been shown to reduce the expression and phosphorylation of STAT1, the central mediator of type I interferon signaling [49]. Interestingly, the suppression of STAT1 expression was found necessary for HPV genome amplification and the maintenance of episomes [49]. E6 and E7 may also induce the expression of leukemia inhibitory factor (LIF), which can repress type I interferon expressed in plasmacytoid dendritic cells (pDCs) and CXCL9 in tumor-associated macrophages, limiting the infiltration of effector CD8^+^ T cells to create an immunosuppressive environment [50]. Tissue tropism between α- and β-HPV types appears to be partially dependent on the L1 capsid protein, which mediates interactions with basal keratinocytes [51]. Mistry et al. reported that cutaneous and mucosal HPV L1 proteins differ in their net charge: α-HPV16 L1 has a net positive charge while the L1 protein of β-HPV5 is net negatively charged [52]. These observations are consistent with the importance of heparan sulfate, a negatively charged cell surface glycosaminoglycan (GAG), in facilitating α-HPV infection in vitro [53,54,55]. Higher GAG expression was found in vaginal tissue compared to the perianal skin, further highlighting how the relationship between viral L1 and GAGs can dictate tissue tropism across HPV types [56].

Langerhans cells (LCs) are a specialized subset of dendritic cells (DCs) strategically placed within the epithelial lining to recognize viral proteins through the expression of PRRs and play a central role in immunoregulation of the cutaneous epithelium, oral, and genital mucosae [57,58]. Like other DCs, LCs can interact with and capture invading viruses using PRRs, migrate to the lymph node, and prime T cells through antigen presentation [59,60]. LCs have been associated with HPV clearance, and the suppression of LC function facilitates HPV persistence [61]. HPV infection has also been shown to disrupt the balance between immunostimulatory and immunoinhibitory states of LCs, favoring the accumulation of LCs with an immunoinhibitory gene signature [62]. Interestingly, basal cell carcinomas and SCCs exhibit a reduced LC presence in the peritumoral and tumoral regions [63]. Tuong et al. also observed the reduced expression of IL-34, a molecule critical for LC homeostasis, in HPV-associated cervical epithelial cancers [62].

While protective B cell responses are crucial for preventing infection, antibodies are not effective at eliminating HPV-infected cells [64,65,66]. It is well-established that T cell (CD4^+^ and CD8^+^) immunity plays an important role in controlling HPV infection at both cutaneous and mucosal tissues (Figure 1) [40]. Following HPV infection, viral clearance and disease regression are driven by CD8^+^ T cells through perforin and granzyme release upon cognate antigen recognition [67,68]. Studies have shown that the number of tumor-infiltrating CD8^+^ T cells may be an important prognostic marker in HPV^+^ head and neck cancer patients, with high CD8^+^ T cell levels correlating with enhanced overall and relapse-free survival [69,70,71,72]. A similar trend has also been observed in HPV-induced cervical cancer [73,74]. Immunohistochemical analysis of cervical biopsies obtained from HPV^+^ and HPV^−^ women showed that epithelial CD8^+^ T cells were substantially reduced in cervical lesions when compared to the HPV-infected normal cervix despite an increase in overall lymphocyte infiltration [75]. In a related study, the prognostic value of intraepithelial tumor-infiltrating lymphocytes (TILs) was investigated in 115 cases of cervical cancer, where a low CD8^+^/regulatory T cell ratio was associated with decreased survival [76]. The importance of CD4^+^ T cells during HPV infection is exemplified by patients with regressed HPV^+^ lesions containing significantly higher numbers of HPV16 E6 and E7-specific CD4^+^ T cells [77,78,79]. Moreover, due to the progressive loss of CD4^+^ T cells, HIV^+^ individuals are more susceptible to HPV acquisition and infection [80]. CD4^+^ T cells with cytotoxic functions have also been described in the context of viral infections, autoimmunity, and cancer, and are especially important when pathogens and tumors downregulate MHC-I to evade CD8^+^ T-cell mediated killing [81,82,83,84]. Little is known about the role of cytotoxic CD4^+^ T cells during HPV infection, as only a few studies have been reported. Facchinetti et al. tested CD4^+^ T cells from 16 healthy donors for reactivity to synthetic HPV18 E6 peptides and recombinant E6 protein and found that CD4^+^ T cells from 3 donors proliferated in the presence of HPV18 E6 antigens and produced IFN-γ, indicating the presence of some HPV-specific CD4^+^ T-cell responses with cytotoxic functions [85]. Furthermore, a key role of the CD28 T cell co-stimulation pathway in controlling common warts caused by HPVs was recently discovered [86,87]. Direct evidence for the immunological control of cutaneous and oral mucosal papillomavirus infection by T cells has been demonstrated in several preclinical models [88,89,90,91,92,93]. Cutaneous infection of immunocompetent mice with MmuPV1 showed strong accumulation of CD4^+^ and CD8^+^ T cells to the challenge site and that T cell depletion prior to MmuPV1 challenge leads to significant growth of papillomas [94,95,96,97]. Strong CD8^+^ T cell responses to E6, and to a lesser extent E7, was described in C57BL/6 mice that had the capacity to clear established cutaneous infections, although CD4^+^ T cells also seemed to impose direct cytotoxicity effects independently of CD8^+^ T cells [98,99]. Collectively, harnessing HPV-specific T-cell responses by vaccination or cell-based therapies may improve clinical benefit for patients with HPV-associated cancers.

Cutaneous HPV types, such as the β-HPVs, are ubiquitous and widespread in the general population [100,101,102]. Guennoun et al. found that β-HPVs generate more immunogenic peptides when compared against both high- and low-risk α-HPV types, which may better complement the host’s human leukocyte antigen (HLA) repertoire [103]. They postulate that this may be a mechanism by which β-HPVs promote higher control by T cell immunity, allowing them to achieve widespread asymptomatic colonization in humans [103]. Interestingly, T-cell immunity against β-HPVs may also suppress skin cancer development in immunocompetent hosts, and it is believed that the loss of this immunity, rather than the oncogenic effect of the virus, causes the increased risk of skin cancer in immunosuppressed individuals [26,104,105]. For instance, Borgogna et al. used HPV8-transgenic *Rag2*-deficient mice to provide insight into the relationship between a compromised immune response, β-HPV infection, and UVB exposure in the development of skin cancer [106]. Exposure to low doses of UVB radiation caused severe skin inflammation that resembled cutaneous field cancerization in organ transplant recipients with enhanced proinflammatory cytokine production and mast cell recruitment to the dermis [106]. Likewise, studies showed that CD8^+^ T-cell depletion increased the risk of MmuPV1-induced cSCC after UVB treatment, indicating the importance of anti-cancer T-cell immunity in this model [107]. While β-HPVs are predominantly associated with cutaneous infections, they may also contribute to infection at mucosal sites [108].

Following pathogen clearance, a subset of effector T cells differentiate into long-lived memory cells, enabling a robust recall response upon subsequent challenge. Tissue-resident memory (T_RM_) T cells play a critical role in host defense by providing rapid, localized protection at barrier sites such as the skin and mucosae, where they cluster at sites of pathogen entry and persistence. Here, T_RM_ cells adapt to the unique microbial and metabolic environment of the tissue, balancing tolerance to commensals with responsiveness against pathogens and contributing to the repair and resolution of inflammation. T_RM_ T-cell responses may be impaired in the presence of HPV infection and in HPV-associated cancers. For instance, high-risk HPV infection has been shown to impede TGF-β signaling through the activity of E5 and E7 [109,110,111], potentially impacting the ability of keratinocytes to retain T_RM_ T cells by downregulating the expression of anchorage protein CD103. Likewise, in the presence of chronic antigen stimulation and under the influence of an immunosuppressive milieu, T_RM_ T cells may become dysfunctional during persistent HPV infection and in HPV-associated cancers [112]. Interestingly, it has also been shown that not all T_RM_ are created equal, as Wang et al. identified six CD8^+^ T_RM_ clusters in single cell sequencing data of normal cervical and cancer tissues that feature a range of cytotoxic and inhibitory marker expression patterns [112]. Furthermore, there may be a preferential localization of subsets within HPV^+^ lesions: T-cell receptor (TCR) sequencing of human ectocervical biopsy specimens revealed two distinct CD8^+^ populations with little overlap between their TCR repertoires [113]. In this dataset, the CD69^hi^CD103^low^IFN-γ^high^Granzyme B^low^ T_RM_ were distributed evenly between the epithelium and the stroma, while the CD69^med^CD103^high^IFN-γ^low^Granzyme B^high^ T_RM_ were preferentially localized in the epithelium. The prognostic benefit of increasing CD8^+^CD103^+^ tumor-infiltrating lymphocytes has been examined in patients with cervical cancer [114]. Here, the association of CD103 expression with clinicopathological variables and outcome was examined in 764 cervical cancer cases and showed that CD103 gene expression was not only correlated with the expression of cytotoxic T cell markers but was also strongly associated with an improved prognosis in patients receiving radiotherapy. Therefore, strategies to boost T_RM_ density and effector function in HPV-infected tissues could be pivotal for next-generation therapeutics. In this regard, “prime and pull” vaccination shows promise to increase T cell numbers at genital mucosal sites and will be discussed in the therapeutic approaches against HPV infections section below.

### 3.3. HPV-Mediated Mechanisms to Evade T Cell Immunity

While most α- and β-HPV infections are transient, a number of immune evasion strategies have been identified that may facilitate viral persistence. Viral protein expression can interfere with TLR signaling pathways, reducing cytokine production and dampening host immune responses [115]. In the vaginal tract, HPV infection has been shown to suppress TLR9 transcription and block pattern recognition receptor (PRR) signal transduction cascades through the activity of E7, decreasing the pro-inflammatory immune response [61,116]. High-risk HPV E7 can also decrease MHC-I expression to impair cytotoxic T lymphocyte (CTL) activation by interacting with the promoter and subsequent recruitment of histone deacetylases [117,118]. Some viral proteins can interfere with viral antigen processing, preventing their presentation by the MHC class I complex [119].

Interestingly, Human Leukocyte Antigen G (HLA-G) is an HLA class I molecule with immunosuppressive properties found on select cells in the peripheral blood, locations of inflammation, and immune-privileged sites such as the cornea, endothelial and erythroid precursors, and thymus [120,121]. HLA-G exerts its immunosuppressive effect through interaction with its cognate receptors on the surface of NK, B, T cells, and APCs and are associated with various cancers and autoimmune disorders [120,122]. Specifically, HLA-G can directly inhibit cell cycle progression, engage inhibitory receptors, and promote an anti-inflammatory profile [123,124,125]. In the context of HPV infection, Ferguson et al. showed that select HLA-G polymorphisms influence the susceptibility to and persistence of high-risk HPV16 and other α-HPV types [126]. The expression of HLA-G may also facilitate immune escape, cervical lesion progression, and malignant transformation in cervical cancer [127].

CD8^+^ T cell responses may also be dampened by regulatory CD4^+^ T cells (Tregs) present within HPV-induced lesions. Tregs have been widely shown to drive HPV infection and HPV-associated malignancies through direct cell-to-cell contact and secretion of immunosuppressive cytokines such as TGF-β and IL-10 [128,129]. The expression of CD25, or interleukin-2 receptor alpha (IL-2Rα), on Tregs can impair the expansion and activation of effector T cells by depriving the milieu of IL-2 [130]. Tregs also express co-inhibitory molecule CTLA-4, which binds CD80 and CD86 on APCs and prevents their interaction with CD28 to block T cell activation [131]. In mice, Treg-specific depletion of CTLA-4 resulted in the development of systemic lymphoproliferation and fatal T cell-mediated autoimmune disease [131]. Furthermore, Treg-secreted TGF-β suppresses the ability of cytotoxic T cells to induce target cell lysis [132,133] and converts naïve CD4^+^ T cells into peripheral Tregs (pTregs) with immunosuppressive function [134]. Conversely, IL-10 predominantly impairs antigen presentation by suppressing TLR signaling, interfering with the IFN-γ-induced activation of macrophages, and downregulating MHC-II and CD86 on dendritic cells, as reviewed elsewhere [135]. A significant increase in Tregs has been observed in both tissues and blood from patients with HPV-induced lesions and cervical cancer [129]. Infected cells have been shown to produce elevated levels of chemokines CXCL12 and CCL22 that may recruit Tregs to HPV^+^ lesions through CXCR4 and CCR4 engagement, respectively [136,137]. Furthermore, patients with metastatic disease may have a higher frequency of Tregs within their lymph nodes [138]. In HPV16^+^ cervical cancer patients, in vitro Treg depletion resulted in increased IFN-γ-mediated T-cell responses against HPV16 E6 and E7 peptides [129]. In addition to Tregs, tumor-associated macrophages and myeloid-derived suppressor cells (MDSCs) present within the tumor microenvironment contribute to tumor progression by inhibiting anti-tumor immune responses [139]. Such cellular and molecular inhibitory mechanisms also limit the efficacy of T-cell therapies by augmenting T cell activation and effector function. Together, the described cellular and molecular inhibitory mechanisms may work to suppress host T-cell immunity against HPV infections and may have implications on T cell-based therapeutic efficacy, as described below.

### 3.4. Imprinting of Natural HPV Infection on the Immune System

After initial encounter with a pathogen, long-lived memory T- and B-cell responses are generated to protect against subsequent exposures. While natural infection with agents such as the varicella-zoster virus typically grant lifelong protection against reinfection, HPV infection frequently clears yet does not always generate sterilizing immunity. Interferon suppression and MHC-I downregulation are among the previously described mechanisms that contribute to this. HPV frequently infects keratinocytes without inducing overt inflammation, suggesting that professional APCs are suboptimally activated to achieve sterilizing immunity against infection. Recruitment of immune cells to infection sites under non-inflammatory conditions may be restricted, impacting the development of memory T cells in the epithelium. However, there are instances where natural HPV infection with a high-risk type provides protection against reinfection with the same and closely related HPV types [140,141]. To determine whether infection with commensal or low-risk HPV types can provide protection against subsequent infection with a different high-risk HPV type, Guennoun et al. used an in silico approach to identify a panel of HPV immunogenic peptides that was predicted to bind MHC-I [103]. They showed that HPV types within the same species (defined as those that share >70% L1 nucleotide homology) shared multiple conserved, homologous peptides whereas less overlap was observed between HPVs across different genus levels (e.g., α-HPVs compared to β-HPVs) [103]. These observations seem to suggest potential T cell cross-reactivity within similar HPV types (e.g., high-risk α-HPV16 and α-HPV18) but low cross-protection between low- and high-risk HPVs (e.g., β-HPV5 and α-HPV16) [26].

## 4. Therapeutic Approaches Against HPV Infection and Associated Malignancies

Treatment of established HPV infections currently focuses on managing symptoms and preventing complications. Papillomas are frequently treated with topical medications such as imiquimod, a TLR7 agonist that stimulates antigen presentation and the production of proinflammatory cytokines, often leading to resolution [142]. Additionally, procedures such as cryotherapy, electrotherapy, and surgery can be used to aid in the removal of warts or papillomas. In HPV-induced malignancies, the cancerous tissue may be surgically removed or treated using radiotherapy and chemotherapy [143]. In addition to these standard-of-care treatments, there has been recent success using novel T cell-based therapies to boost anti-cancer immunity, paving the way for possible combination treatments against HPV-associated malignancies [144].

### 4.1. Prophylactic and Therapeutic Vaccines

Prophylactic HPV vaccines prime the immune system prior to viral exposure, allowing for a rapid secondary response should the individual subsequently encounter the virus. Currently, there are two prophylactic vaccines approved for administration in Canada, Gardasil^®^ 9, and Cervarix. Gardasil^®^ 9 is a recombinant HPV vaccine that offers protection against 9 low- and high-risk α-HPV types, preventing >95% of mucosal infections and precancerous lesions [145,146,147]. Cervarix is a recombinant, bivalent vaccine that offers protection against >90% of persistent infections and cervical lesions caused by HPV16 and HPV18 infections [148,149]. Both vaccines are comprised of recombinant L1 proteins that self-assemble into virus-like particles (VLPs) upon injection and induce high affinity polyclonal anti-L1 IgG antibody responses [150,151]. These vaccines have also been shown to increase the frequency of HPV-specific CD4^+^ T cells [152]. Cervarix may elicit a stronger and more sustained cell-mediated response due to its unique adjuvant, AS04, which stimulates Th1-type cellular responses [153]. To understand why these vaccines are strictly preventative and not therapeutic, we should consider the HPV life cycle and the specific immunological pathways these vaccines activate. The L1 protein is expressed on extracellular virions and mediates viral entry into host cells. As such, prophylactic vaccine-induced anti-L1 antibodies neutralize the virus before it enters host cells. Once the HPV genome becomes internalized, however, it primarily expresses early proteins E6 and E7. Here, cytotoxic T lymphocytes recognize these viral peptides presented on MHC-I and trigger apoptosis in the infected cells. This prompts the need for therapeutic vaccines that elicit cell-mediated immunity to clear virally infected cells and associated malignancies.

There are many therapeutic vaccine platforms currently under investigation, with the majority aiming to induce CD4^+^ and CD8^+^ T-cell responses against high-risk HPV oncogenes E6 and E7. A recent Phase II trial to evaluate the clinical benefit of Vvax001, a replication-deficient recombinant Semliki Forest virus (rSFV) encoding HPV16 E6 and E7, showed improved HPV16 clearance and facilitated histopathologic regression in women with HPV16^+^ cervical intraepithelial neoplasia (CIN)-3 lesions [154,155]. Vaccination induced measurable E6- and E7-specific type I cytokine responses by both CD4^+^ and CD8^+^ T cells and the formation of long-lasting HPV-specific memory T cells, confirmed by responses up to 6 months after the start of treatment. Likewise, Jiang et al. showed that vaccination of MmuPV1-challenged SKH-1 mice with DNA hCRT-mE6mE7mL2 induced a strong E6/E7-specific CD8^+^ T-cell immune response sufficient to drive papilloma clearance [95]. Adoptive transfer of E6-specific CD8^+^ T cells also led to the regression of MmuPV1-induced lesions in another study [94]. However, many current therapeutic vaccines against high-risk HPV have modest efficacy in regressing precancerous lesions (54% regression of CIN-2/3 lesions to normal/CIN-1 across 12 trials involving 734 women) when compared against standard-of-care excision techniques (90% regression) [156]. Poor vaccine stability, low immunogenicity, risk of adverse reactions, and heterogeneity across platforms and endpoints are among the reasons why therapeutic HPV vaccines have yet to be approved for widespread use [157]. Current therapeutic vaccines mainly target the E6 and E7 antigens of HPV and fail to overcome the immunosuppressive microenvironment established by the virus during persistent HPV infections. Therefore, prospective vaccination efforts should incorporate new therapeutic targets (e.g., focus on other conserved early genes) and aim to overcome the immunosuppressive environment of cancer lesions. One such approach is the potential use of drugs that induce Treg fragility in combination with immune checkpoint blockade therapy, thereby improving anti-tumor T-cell priming and tumor control [158,159]. The success of therapeutic vaccines may also depend on their delivery platform, of which the DNA-based vaccines have shown promise [160,161]. DNA-based vaccines induce cellular and humoral immune responses and offer practical advantages over other vaccines platforms, including the potential for rapid and scalable manufacturing, targeting of multiple antigens, and thermostability over extended periods of time.

Recently, the “prime and pull” strategy of vaccination was proposed and studied in the context of herpes simplex virus 2 (HSV-2) and severe acute respiratory syndrome coronavirus 2 (SARS-CoV-2) infection [162,163,164]. Effective recruitment of immune cells to sites of mucosal infection, such as the vaginal tract, may be restricted under non-inflammatory conditions. The “prime and pull” strategy has shown promise in reducing the spread of infection and preventing recurrent symptoms caused by mucosal viruses by promoting immune cell infiltration. First, a conventional intramuscular vaccination is administered to “prime” naïve T cells. Next, a cream is applied to the skin that contains a medication to “pull” T cells to the mucosal site. This method establishes a pool of resident memory T cells within tissues where T cell travel and entry may be restricted [162,163,164]. Development of this approach may boost T-cell immunity and provide long-lasting protection against reinfection at genital mucosal sites.

### 4.2. Engineered T Cells

Engineered T-cell therapy has shown tremendous promise in the treatment of B-cell malignancies and is being investigated as a therapeutic approach for a wide range of viral and nonviral cancers. Here, T cells are genetically modified to express a defined T-cell receptor (TCR) on their surface that is capable of targeting a specific MHC-presented antigen [165]. Viral antigens, such as HPV16 oncoproteins E6 and E7, are viable targets for engineered T-cell effector function due to their restricted expression in infected or transformed tissues [166,167]. In a Phase I/II clinical trial, E7-primed TCR-T cells showed robust tumor regression in 6 out of 12 patients with metastatic HPV16^+^ epithelial cancers [168]. Eleven patients with squamous cell carcinomas and 1 patient with an adenocarcinoma that were primarily localized to the uterine cervix (*n* = 5), head and neck (*n* = 4), anus (*n* = 2), and vulva (*n* = 1) were recruited and treated with T cells expressing the E7 TCR and aldesleukin, a recombinant form of IL-2. The ability of adoptively transferred E6-primed TCR-T cells to target chemotherapy-refractory, metastatic HPV16^+^ epithelial cancer was also studied [169]. Nine patients with SCCs (3 cervical, 1 vaginal, 4 anal, and 1 head and neck) and 3 patients with cervical adenocarcinomas that had received prior platinum-based therapy were enrolled in this study and treated with autologous genetically engineered T cells alongside a conditioning regimen and systemic aldesleukin. However, despite the use of a high-avidity TCR against a constitutively expressed oncoprotein, the response rate was modest, with only 2 out of 12 patients showing tumor regression one month following treatment [169]. Alternatively, chimeric antigen receptor (CAR) T cells that link an extracellular antigen-binding domain to intracellular signaling domains offer the potential to target any cell surface antigen without the need for MHC antigen presentation, provided that antigen density and tumor specificity are sufficient [170]. While CAR T cells have achieved clinical success in the treatment of B-cell malignancies, their broader applicability for virus-associated cancers remains limited due to a lack of targetable surface antigens, impaired trafficking and infiltration through the tumor microenvironment, and toxicity in patients [170,171].

### 4.3. Immune Checkpoint Blockers

Immune checkpoint blockade (ICB) therapies have also been explored in the treatment of HPV-associated cancers. In ICB therapy, monoclonal antibodies are used to block immune checkpoint proteins such as programmed cell death protein 1 (PD-1), programmed death ligand 1 (PD-L1), and cytotoxic T lymphocyte antigen 4 (CTLA-4), inhibiting their immune dampening effects. Eberhardt et al. studied CD8^+^ T-cell responses in patients with HPV^+^ head and neck cancer and identified a population of HPV-specific PD-1^+^TCF-1^+^CD45RO^+^ stem-like CD8^+^ T cells that may expand and differentiate into a pool of effector CD8^+^ T cells capable of killing virally infected cells when treated with a PD-1 blocker [172]. ICB therapies have shown promise in the treatment of both locally advanced and metastatic cervical cancers. In the KEYNOTE-826 phase 3 clinical trial, the combination of pembrolizumab with chemotherapy showed a significant improvement in overall survival in patients with persistent, recurrent, or metastatic cervical cancer [173]. Similarly, an increase in overall and progression-free survival was observed in the treatment of locally advanced cervical cancer with pembrolizumab and concurrent chemotherapy in the KEYNOTE-A18 phase 3 trail [174]. ICB therapy shows some clinical benefit in the treatment of HPV^+^ head and neck squamous cell carcinoma (KEYNOTE-048 trial) and anal squamous cell carcinoma (POD1UM-303 trial) [175,176]. However, the universal application of ICB for HPV-associated cancers remains limited due to mechanisms of HPV-mediated immunosuppression, the complex tumor microenvironment, immune-related adverse events following treatment, and high cost. A recent study demonstrated that HPV-positive anogenital SCCs are associated with a decreased PD-L1 expression compared against HPV-negative tumors [177]. Therefore, in addition to PD-1 and PD-L1, HLA-G may be an important target for immune checkpoint inhibition [178,179,180]. Antibodies and small molecule inhibitors that block the interaction between HLA-G and its receptors are also currently under investigation as cancer immunotherapy [127,178]; however, further studies are needed to validate these early results.

## 5. Conclusions and Future Directions

**Figure 2:**
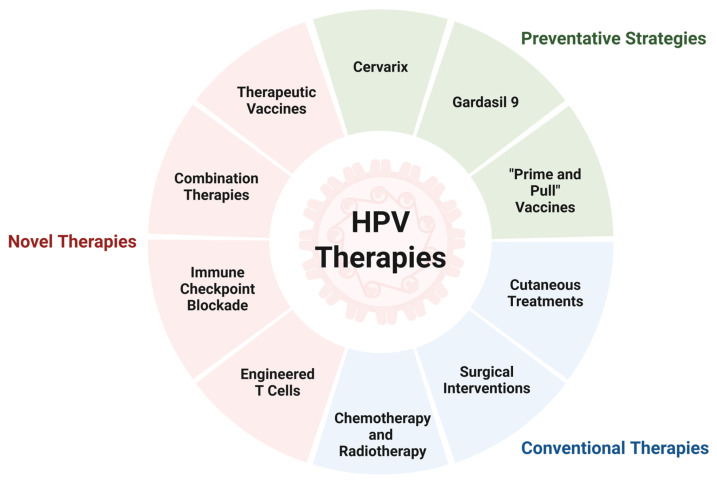
Therapeutic landscape for the treatment of HPV infection and associated malignancies. Several preventative strategies (green), and conventional (blue) and novel HPV therapies (red) are outlined above. Among the preventative approaches, in addition to FDA-approved vaccines, such as Gardasil 9 and Cervarix, the “prime and pull” vaccines show promise in their ability to recruit primed T cells to the mucosa. Further, unlike conventional HPV therapies, many novel therapies are aimed at enhancing the anti-viral immune response, with the T cell response being a primary research focus. Here, combinatory approaches have yielded the most promising and robust results. *Created in BioRender. Savage, D. (2026). BioRender.com/9kkruce*.

HPV is a prevalent viral infection known for its ability to evade the immune response and persist within the host, thereby increasing the risk of developing cutaneous and mucosal lesions and malignancies. Accumulating evidence indicates that host T-cell responses play a critical role in controlling HPV infections, as demonstrated in a murine model of papillomavirus (MmuPV1) infection. During natural infection, CD8^+^ cytotoxic T lymphocytes are crucial for eliminating HPV-infected cells, suggesting that therapeutic vaccines capable of generating protective anti-cancer CTL responses have the potential for treating HPV-associated diseases. However, HPV-mediated downregulation of MHC-I can impair CTL responses. This limitation may be overcome by using genetically engineered cell surface receptors, such as CAR T cell therapies, which are not MHC-restricted. Further investigations into the role of HPV-specific CD4^+^ T cells with cytotoxic functions are needed to evaluate their potential therapeutic role in HPV^+^ cancers. The suppressive role of CD4^+^ regulatory T cells is well-established, and their accumulation in lesions remains a barrier to effective cell-based therapies. The “prime and pull” strategy may be particularly relevant for enhancing long-lasting immunity across the genital mucosa, though it has yet to be tested in the context of HPV infections. Furthermore, near-term priorities should include defining correlates of protection in tissue, improving antigen delivery to generate tissue-resident memory T cells, and biomarker-guided patient selection. In conclusion, a clear mechanistic understanding of protective immunity along with the immune evasion strategies employed by HPV can be leveraged to develop vaccine-based therapies against HPV^+^ cancers, with several approaches showing promising clinical benefit (Figure 2).

## Figures and Tables

**Table 1:** Summary of key biological and clinical characteristics of alpha- and beta-HPVs. Differences in tissue tropism, infection types, risk factors of disease progression, mechanisms of oncogenesis, and transmission have been highlighted.

Feature	Alpha-HPVs	Beta-HPVs
Viral Types	Low-risk (e.g., HPV6, HPV11) and high-risk (e.g., HPV16, HPV18) types, segregated based on their ability to drive malignant transformation of infected tissue	Predominantly low-risk types (e.g., HPV5, HPV8)
Tissue Tropism	Predominantly mucosal, e.g., anogenital and oropharyngeal tracts	Cutaneous
Host DNA Interaction	Viral DNA often integrates	Viral DNA often remains episomal
Infection Types	May cause transient, acute, or persistent infections	Commensal or pathogenic in nature
Risk Factors of Disease Progression	Viral type, viral load, prolonged use of oral contraceptives, and high parity	Environmental factors and host immune status
Clinical Impact of Progression	Genital warts, mucosal cancers	Cutaneous lesions and warts, skin cancers, e.g., cutaneous squamous cell carcinoma
Mechanism of Oncogenesis	Expression of viral oncoproteins upon entry into basal epithelial cells disrupts cellular apoptosis, differentiation, and DNA repair systems to ensure continuous cell proliferation and replication of the HPV genome, driving malignant transformation.	Promote cellular proliferation in the presence of constant stress, e.g., UV radiation, enabling the accumulation of DNA damage. When the genes of β-HPV-targeted cellular pathways acquire mutations, growth of cancerous cells becomes independent of viral oncogene expression.
Transmission	Direct contact (sexual transmission), perinatal transmission	Skin-to-skin contact and through the environment

**Table 2:** Features that distinguish HPV infection states. This table highlights the participation of alpha- and beta-HPVs in transient, acute, persistent, or commensal infection processes and the role of the immune response in controlling them.

Feature	Transient	Acute	Persistent	Commensal
Typical Genus	Low-risk α-HPV	Low- or high-risk α-HPV	High-risk α-HPV	β-HPV
Duration	Short-term	Short-term, typically months to years	Long-term, typically years to decades	Indefinite
Immune Response	Effective clearance by the host immune response	Active inflammation and viral replication	Immune evasion	Immune tolerance
Clinical Presentation	Asymptomatic	Visible warts or papillomas	Precancerous lesions (e.g., CIN)	None
Viral Load	Decreases over time	High	High	Low and stable
Oncogenic Risk	Negligible	Low	High	Low, unless individual is immunosuppressed

## Data Availability

No new data were created or analyzed in this study. Data sharing is not applicable to this article.
